# The Role of NKL Homeobox Genes in T-Cell Malignancies

**DOI:** 10.3390/biomedicines9111676

**Published:** 2021-11-12

**Authors:** Stefan Nagel

**Affiliations:** Department of Human and Animal Cell Lines, Leibniz-Institute DSMZ, 38124 Braunschweig, Germany; sna@dsmz.de; Tel: +49-531-2616-167

**Keywords:** homeobox, homeodomain, NKL-code, T-all, lymphoma

## Abstract

Homeobox genes encode transcription factors controlling basic developmental processes. The homeodomain is encoded by the homeobox and mediates sequence-specific DNA binding and interaction with cofactors, thus operating as a basic regulatory platform. Similarities in their homeobox sequences serve to arrange these genes in classes and subclasses, including NKL homeobox genes. In accordance with their normal functions, deregulated homeobox genes contribute to carcinogenesis along with hematopoietic malignancies. We have recently described the physiological expression of eleven NKL homeobox genes in the course of hematopoiesis and termed this gene expression pattern NKL-code. Due to the developmental impact of NKL homeobox genes these data suggest a key role for their activity in the normal regulation of hematopoietic cell differentiation including T-cells. On the other hand, aberrant overexpression of NKL-code members or ectopical activation of non-code members has been frequently reported in lymphoid and myeloid leukemia/lymphoma, demonstrating their oncogenic impact in the hematopoietic compartment. Here, we provide an overview of the NKL-code in normal hematopoiesis and discuss the oncogenic role of deregulated NKL homeobox genes in T-cell malignancies.

## 1. T-Cell Development

All blood and immune cells are classified as lymphoid or myeloid and generated by hematopoietic stem cells (HSC) which reside in the bone marrow. The lymphoid lineage begins with the HSC-derived common lymphoid progenitor (CLP). The panel of mature lymphoid cell types generated in the course of lymphopoiesis includes B-cells, T-cells, NK-cells and innate lymphoid cells (ILCs). T-cells derive from early T-cell progenitors (ETP) which migrate from the bone marrow into the thymus to terminate their basic differentiation. Intermediate stages of developing T-cells are phenotypically distinguished by their surface markers CD4 and CD8 and accordingly called double negative (DN), double positive (DP) and single positive (SP) thymocytes. The thymic differentiation processes result in naïve T-cells, possessing selected T-cell receptors able to detect non-self antigens. Further differentiation of T-cells takes subsequently place in extrathymic tissues. 

These fundamental T-cell differentiation processes are mainly regulated at the transcriptional level [1,2]. Accordingly, several hematopoietic and T-cell specific transcription factors (TF) are reported which control the progression of T-cell development. Frequently, their deregulation and mutation underlie the generation of T-cell malignancies [1,3,4]. Therefore, the knowledge and functional understanding of normal and aberrant activities of T-cell specific TFs may help to diagnose and treat T-cell leukemias and lymphomas. 

## 2. NKL Homeobox Genes in Normal and Malignant T-Cells

### 2.1. Classification of Homeobox Genes 

TFs are classified according to similarities in sequence and structure. Homeobox genes encode one of the largest groups of TFs in the human genome [5]. Generally, they regulate basic development and differentiation in both embryogenesis and adulthood. These genes share the conserved homeobox which is 180 bp long and encodes the homeodomain at the protein level. This domain consists of 60 amino acid residues which form three helices, generating a specific 3D structure of the helix-turn-helix type. The homeodomain performs specific interactions with DNA, chromatin, non-coding RNAs and cooperating TFs, thus representing the core of their gene regulatory activities [6]. Specific DNA contacts are realized by helix 3 which fits into the major groove [7]. The remaining helices stabilize the domain structure and, together with flanking amino acid residues, allow additional DNA interactions. 

From the systematic classification of all 235 human homeobox genes emerged a panel of eleven classes and several subclasses. The main classes are called antennapedia (ANTP) and paired box (PRD). Other classes identified are CERS, CUT, HNF, LIM, POU, PROS, SINE, TALE, and ZF. According to this system, NKL homeobox genes represent a subclass of the ANTP class and number 48 members in humans [8]. 

Nirenberg and Kim (abbreviated as NK) were the first who reported in the fruit fly *Drosophila* NK-like homeobox genes which were later summarized as NKL. In this developmental model organism, the NKL subclass members are arranged in a cluster consisting of six genes [9]. Additional orthologous genes were later identified, extending this group of genes. Comparative genome analyses revealed this clustering to be the ancient gene order which remains barely discernible in vertebrates [10]. Thus, human NKL homeobox genes show only relicts of a clustered arrangement while the HOX-genes are still present in a clustered order in both fruit fly and humans. 

**Figure 1:**
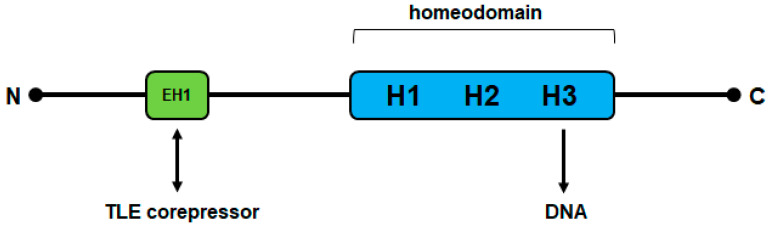
Schematic structure of NKL homeodomain proteins. N: N-terminal end; C: C-terminal end; EH1: conserved engrailed homology domain consisting of about 8 amino acid residues (shown in green); the conserved homeodomain consists of 60 amino acid residues which generate three helices (H1, H2, H3) and is shown in blue; the N- and C-terminal parts show no sequence conservation. The EH1 domain and the homeodomain interact with particular components of the gene regulatory machinery including corepressors of the TLE family and specific DNA sequences, respectively.

NKL proteins share a conserved homeodomain which displays the amino acid tyrosine at position 54 [11]. In addition, NKL homeodomain proteins contain a short conserved sequence in their N-terminal region which has been termed engrailed-homology motif (EH1) [12]. This EH1 sequence mediates physical interactions with corepressors of the Groucho-family (transducin-like enhancer of split, TLE), thus transforming NKL homeodomain factors into transcriptional repressors [13] (Figure 1). Most NKL homeobox genes are involved in mesodermal development, possibly recapitulating their ancient functions, including repression of alternative differentiation lineages [14,15]. Many NKL homeobox genes operate as master factors, further demonstrating the regulatory and developmental potential of these genes. For example, *NKX2-5* controls the development of the spleen and heart, and *NKX2-1* that of lungs and the thyroid gland [16,17].

### 2.2. NKL-Code in Hematopoiesis

**Figure 2:**
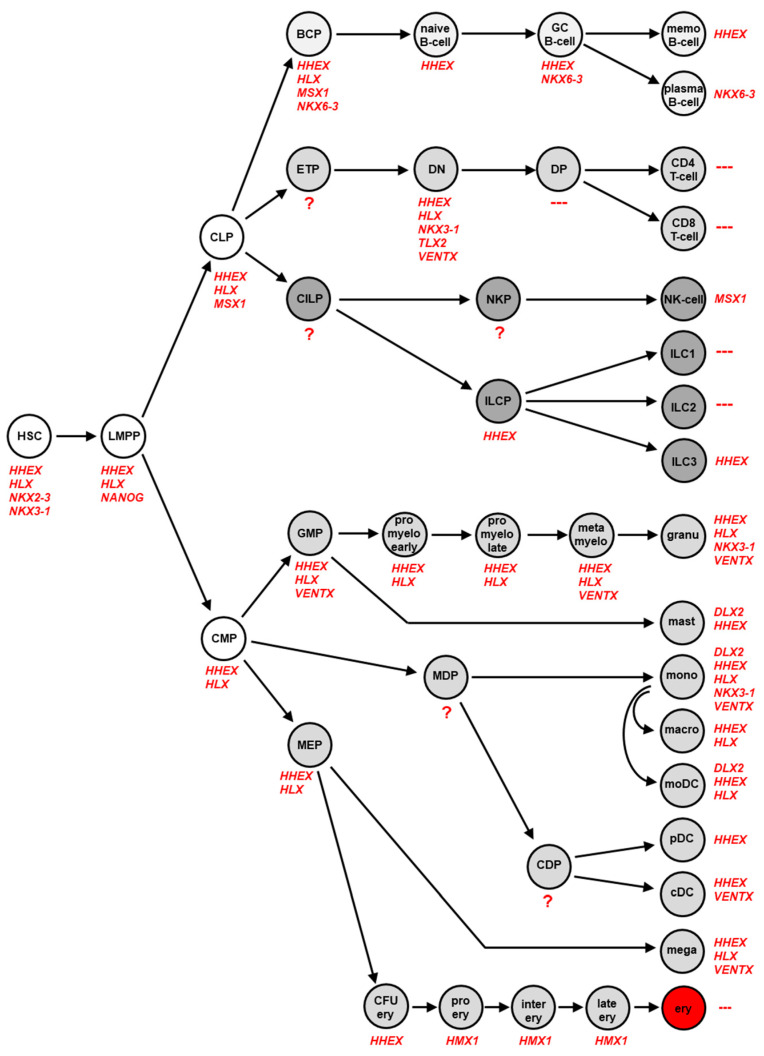
The NKL-code in lymphopoiesis This diagram depicts activities of NKL homeobox genes during early hematopoiesis, in lymphopoiesis including development of T-cells, B-cells, NK-cells and ILCs, and in myelopoiesis. Each cell-type/stage is labelled with the accordingly expressed NKL homeobox genes. BCP: B-cell progenitor, cDC: conventional dendritic cell, CDP: common dendritic progenitor, CILP: common innate lymphoid progenitor, CLP: common lymphoid progenitor, CMP: common myeloid progenitor, DN: double negative, DP: double positive, ETP: early T-cell progenitor, GC B-cell: germinal center B-cell, GMP: granulocytic-monocytic progenitor, HSC: hematopoietic stem cell, ILC(P): innate lymphoid cell (progenitor), LMPP: lymphoid and myeloid primed progenitor, memo B-cell: memory B-cell, MEP: megakaryocyte-erythroid-progenitor, pDC: plasmacytoid dendritic cell. ?: no expression data were available for progenitor stages CLP, NKP, MDP and CDP, ---: no NKL homeobox gene activity detectable.

In several studies, we examined the physiological expression pattern of NKL homeobox genes in stem cells, intermediate progenitors and terminally differentiated blood and immune cells across the whole hematopoietic system. The lymphoid lineage was analyzed in four studies using public datasets of developing and mature T-cells, B-cells, NK-cells and ILCs [18,19,20,21]. Datasets covering the myeloid lineage were analyzed in two studies [22,23]. The accumulated results are summarized in Figure 2, showing specific NKL homeobox gene activities in distinct hematopoietic stages and cell types. Altogether, eleven NKL homeobox genes were identified, comprising *DLX2*, *HHEX*, *HLX*, *HMX1*, *MSX1*, *NANOG*, *NKX2-3*, *NKX3-1*, *NKX6-3*, *TLX2* and *VENTX*. Their unique expression pattern has been termed NKL-code [18,24]. 

The most prominent representatives of the NKL-code are *HHEX* and *HLX* active in HSC, multiple progenitors, and various mature blood cells. In contrast, the expression of *NKX2-3* and *NANOG* is restricted to the earliest stages of hematopoiesis, including HSC and lymphoid and myeloid primed progenitors (LMPP), respectively. *NKX6-3* is just expressed in the B-cell lineage, *TLX2* in the DN-stage of T-cell development, *DLX2* in mature mast cells and monocytes, and *HMX1* in the course of erythropoiesis. *MSX1* is expressed in CLP and in NK-cells. *NKX3-1* shows a diverse expression pattern comprising undifferentiated HSC and DN T-cells in addition to differentiated granulocytes and monocytes. Recently, *VENTX* expression was described in progenitor-derived conventional dendritic cells (DC) while silent in plasmacytoid DC [23]. Of note, *VENTX* is also expressed at the DN stage of developing T-cells [18]. An absence of NKL homeobox gene activity was found for DP, CD4 and CD8 SP T-cells and for ILC1 and ILC2. Thus, in contrast to these lymphoid cell types remaining hematopoietic cells specifically express one or several members of the NKL homeobox gene subclass. DN T-cells express five different NKL homeobox genes while mature T-cells are silent for all subclass members. This observation may indicate that accurately controlled activity of NKL homeobox genes plays an important role in developing T-cells which are, therefore, prone to malignant transformation when these genes are deregulated. 

Of note, some screenings for gene activities in these various hematopoietic cell types were conducted by expression profiling. The used standard gene chips do not contain the complete panel of known human genes and lack eleven NKL homeobox genes. However, analysis of RNA-seq data generated from various hematopoietic stem and progenitor cells confirmed inactivity of these eleven genes in immature stages [25], supporting the integrity of the described NKL-code. 

These findings were promoted by data reported for particular NKL homeobox genes and hematopoietic cell types. *HLX* and *HHEX* represent the first described non-HOX homeobox genes expressed in hematopoietic cells [26,27]. Expression analyses of both genes revealed activity in B- and myeloid cells while T-cells tested negative [26,28,29,30]. Moreover, downregulation of *HHEX* was shown to be crucial for normal T-cell differentiation and its activity absent in plasma cells [30]. Analysis of *HHEX*-knockout mice showed disturbed development of all types of lymphocytes, demonstrating the importance of *HHEX* for lymphopoiesis [31]. Recently, a role of *HHEX* was shown in the development of memory B-cells, supporting its reported expression according to the NKL-code [19,32]. Forced expression of *HLX* in hematopoietic progenitors enhanced myeloid differentiation but arrested the development of B-cells at the pro-B-cell stage and of T-cells at the DP stage, highlighting the shutdown of its activity for lymphocyte maturation [28,33]. NKL homeobox genes *HHEX*, *NKX6-3* and *VENTX* have been analyzed for their physiological regulation in hematopoiesis [19,23,34]. The data show that these genes are controlled by known hematopoietic master factors and thus part of lineage-specific gene networks. 

Homeobox genes regulate basic processes in tissue and organ development. This potential, in addition to specific activities of closely related homeobox genes, has been referred to by the annotation of codes. The HOX-code describes the ordered expression of the clustered HOX genes along the anterio-posterio axis of the developing hindbrain and of the embryonal pharynx [35,36]. The DLX-code addressed the expression pattern of DLX genes along the dorsal-ventral axis in the pharyngeal region of the embryo [37]. The identity of the developing placodes which generate different sensory organs is manifested by the expression of particular PAX genes and, accordingly, termed PAX-code [38]. Finally, the TALE-code defines a signature of TALE class homeobox genes in lymphopoiesis [39]. Thus, the NKL-code along with other homeobox gene codes serves to outline and understand developmental gene activities and their subordinate differentiation processes. 

### 2.3. Deregulated NKL Homeobox Genes in T-Cell Acute Lymphoid Leukemia

In cell and tissue differentiation, specific intermediate stages are distinguishable. In cancer, normal progression is disturbed and developmental arrest at particular immature stages is a dominant and widespread feature of malignant cells [40,41,42]. Thus, cancer may represent a developmental disease. Accordingly, forced expression of NKL homeobox gene HLX in hematopoietic progenitors resulted in the developmental arrest of pro-B-cells [33]. This experiment was the first hint for the oncogenic potential of NKL homeobox genes in hematopoietic cells. Later, Ferrando and coworkers described the aberrant expression of NKL homeobox gene TLX1 at the DP stage in T-cell acute lymphoid leukemia (T-ALL), indicating that this gene specifically promotes developmental arrest of malignant thymocytes [4]. The strong correlation of aberrantly expressed NKL homeobox genes with particular stages of lymphoid differentiation highlights the developmental potency of these genes in hematopoietic tumors. 

**Table 1:** Aberrantly expressed NKL homeobox genes in T-cell leukemia/lymphoma patients and cell lines.

Gene	AITL	ALCL	ATLL	HSTL	NKTL	PTCL	T-ALL	T-Cell Line Models	References
***BARHL1***							+		
***BARHL2***									
***BARX1***									
***BARX2***							+		
***BSX***									
***DBX1***									
***DBX2***									
***DLX1***							+		
***DLX2***							+	HPB-ALL	[43]
***DLX3***							+		
***DLX4***					+				
***DLX5***									
***DLX6***							+		
***EMX1***									
***EMX2***							+		
***EN1***						+			
***EN2***						+			
***HHEX***	+	+	+	+	+	+	+	CCRF-CEM, RPMI-8402	[43]
***HLX***	+	+	+	+	+	+	+	DEL, KI-JK, L-82, SR-786, SU-DHL-1, SUP-M2	[21]
***HMX1***									
***HMX2***					+				
***HMX3***									
***LBX1***							+		
***LBX2***							+		
***MSX1***	+	+	+	+		+	+	DERL-2, DERL-7, LOUCY	[44,45]
***MSX2***							+		
***NANOG***					+				
***NKX1-1***	+	+				+	+		
***NKX1-2***									
***NKX2-1***	+	+	+			+	+	RPMI-8402	[43]
***NKX2-2***	+	+	+			+	+		
***NKX2-3***	+	+	+	+	+	+	+		
***NKX2-4***									
***NKX2-5***		+		+	+	+	+	CCRF-CEM, PEER	[46]
***NKX2-6***									
***NKX2-8***									
***NKX3-1***					+		+	JURKAT, MOLT-4, RPMI-8402	[47]
***NKX3-2***							+	CCRF-CEM	[48]
***NKX6-1***		+				+			
***NKX6-2***									
***NKX6-3***					+		+		
***NOTO***									
***TLX1***				+			+	ALL-SIL	[49]
***TLX2***							+		
***TLX3***					+		+	DND-41, HPB-ALL	[50]
***VAX1***									
***VAX2***					+				
***VENTX***									
48	7	9	6	6	11	11	25		

This table lists all 48 NKL homeobox genes and shows deregulated subclass members in defined T-cell malignancies. (marked with ”+”). NKL-code members are indicated in red letters, genes not represented on standard expression arrays are indicated in blue letters. Angioimmunoblastic T-cell lymphoma (AITL), anaplastic large cell lymphoma (ALCL), adult T-cell leukemia/lymphoma (ATLL), hepatosplenic T-cell lymphoma (HSTL), NKT-cell lymphoma (NKTL), peripheral T-cell lymphoma (PTCL), and T-cell acute lymphoid leukemia (T-ALL). NKL-positive T-cell malignancies are marked with “+”.

The NKL-code allows the evaluation of NKL homeobox gene activities in hematopoietic malignancies. Table 1 summarizes reported deregulated NKL homeobox genes identified in T-cell malignancies, including angioimmunoblastic T-cell lymphoma (AITL), anaplastic large cell lymphoma (ALCL), adult T-cell lymphoma (ATLL), hepatosplenic T-cell lymphoma (HSTL), natural killer T-cell leukemia/lymphoma (NKTL), peripheral T-cell lymphoma (PTCL) and T-ALL [44]. Together, 32 aberrantly expressed genes of the 48 gene strong NKL homeobox gene subclass have been described in these cancers, demonstrating their ubiquity and oncogenic impact in the T-cell compartment. Despite their relatively recent discovery, NKL homeobox genes form the most substantial group of oncogenes in leukemia and lymphoma. With few exceptions, most deregulated NKL homeobox genes are expressed in both lymphoid and myeloid malignancies. As analyzed so far, aberrantly expressed NKL homeobox genes show no mutations in their coding regions. Mutations in non-coding regulatory regions have been reported for *HMX2* and *HMX3* in acute myeloid leukemia (AML), highlighting transcriptional deregulation as a main pathological cause [51]. The following account summarizes deregulating mechanisms and oncogenic functions of selected NKL homeobox genes hitherto described in T-ALL. 

#### 2.3.1. TLX1

In 1991, investigation of chromosomal rearrangement t(10;14)(q24;q11) in T-ALL patients by the group of Stanley Korsmeyer demonstrated juxtaposition of T-cell receptor gene *TRD* and T-cell leukemia homeobox 1 (*TLX1*, formerly *HOX11*) [49]. Since then, aberrantly activated *TLX1* emerged as a hallmark oncogene for this malignancy, although *TLX1* is expressed in merely 10% of pediatric and 30% of adult T-ALL patients [50]. Retrospectively, *TLX1* was the first NKL homeobox gene reportedly deregulated in hematopoietic malignancies. Normally, *TLX1* plays a fundamental role in early spleen development, though it is not expressed in hematopoietic cells [52]. Therefore, *TLX1* may perform to some extent its oncogenic function by reactivation of embryonal splenic activities. 

#### 2.3.2. TLX3

In 2001, the group of Roger Berger reported a novel cryptic translocation, t(5;14)(q35;q32), in T-ALL subsets. This aberration activates T-cell leukemia homeobox 3 (*TLX3*, formerly *HOX11L2*) by juxtaposition to the *BCL11B* locus and was detected in about 25% of pediatric and 5% of adult T-ALL patients [53]. Despite their high similarity, *TLX1*- and *TLX3*-rearranged T-ALL patients show different prognoses [54]. Functionally, T-cell differentiation factor *BCL11B* is downregulated by chromosomal juxtaposition with *TLX3* or directly by TF TLX1 [55], highlighting its tumor suppressor activity in T-ALL. Furthermore, TLX1 and TLX3 interact with TF ETS1 in T-ALL cells, thereby blocking TCR-rearrangement and T-cell differentiation [56]. Another gene family member, *TLX2* (*HOX11L1*), though physiologically expressed in T-cell progenitors, is only rarely deregulated in T-ALL [18].

#### 2.3.3. NKX2-5

In 2003, we reported an alternative, cytogenetically identical t(5;14)(q35;q32) in two T-ALL cell lines which juxtaposes *BCL11B* with NK2 homeobox 5 (*NKX2-5*, formerly *CSX1*) [46]. This indicative finding helped us to uncover the wider oncogenic role of NKL homeobox genes in T-ALL, then comprising *TLX1*, *TLX3* and *NKX2-5*. Today, 24 deregulated NKL homeobox genes are described in T-ALL, supporting the importance of this group of oncogenes for this disease [18,57]. However, *NKX2-5* is rarely expressed in this malignancy [18,58]. Normally, *NKX2-5* plays a key physiological role in the development of the spleen and heart [16,51]. Uniting its physiological and leukemic roles, myocyte-specific enhancer factor 2C (*MEF2C*) has been shown to serve as a target gene of *NKX2-5* in both heart and leukemic T-cells [59,60]. *MEF2C* itself has, meanwhile, emerged as a major oncogene in T-ALL, alternatively activated by chromosomal deletion and translocation, or by other deregulated transcription factors [60,61,62]. 

#### 2.3.4. NKX2-1

In T-ALL, chromosomal translocation t(7;14)(q34;q13) causes aberrant activation of NK2 homeobox 1 (*NKX2-1*) [61]. *NKX2-1*, like *NKX2-2* and *NKX2-5*, activates *MEF2C* in T-ALL [61]. In addition, NKX2-1 is aberrantly activated in diffuse large B-cell lymphoma (DLBCL) where it is deregulated by an altered chromatin configuration instead of a chromosomal rearrangement [63]. Thus, the same NKL homeobox gene is aberrantly activated by diverse mechanisms in different lymphoid malignancies. Normally, *NKX2-1* plays a role in the development of the lung and thyroid gland but is absent from hematopoietic cells and tissues [17].

#### 2.3.5. NKX2-3

Recently, a cytogenetic screen for novel translocation partners of the TCR genes in T-ALL revealed targeted NKL homeobox gene *NKX2-3* [57]. In the hematopoietic compartment, *NKX2-3* is expressed only in HSCs [18]. Therefore, aberrant activation of this hematopoietic stem cell gene may deregulate or stop normal T-cell differentiation. 

#### 2.3.6. NKX2-4

Another cytogenetic screen for TCR translocation partners in T-ALL revealed juxtaposition to *NKX2-4* [64]. This NKL homeobox gene is no NKL-code member and thus silent in hematopoietic cells. Normally, *NKX2-4* is just expressed in the hypothalamus [65,66]. Its oncogenic role in T-cell malignancies is hitherto unclear. 

#### 2.3.7. NKX3-1

NK3 homeobox 1 (*NKX3-1*, formerly *BAPX2*) is hematopoietically expressed in stem cells and DN T-cell progenitors [18]. In T-ALL, *NKX3-1* is aberrantly activated by the hematopoietic TFs TAL1 and GATA3 [47,67]. *NKX3-1* expression correlates with oncogenic TAL1 activity and additionally with aberrant expression of homeobox gene *SIX6* in T-ALL patients [68]. Consistently, *SIX6* in turn is directly activated by *NKX3-1* or alternatively by the closely related NKL-factor *NKX3-2* (*BAPX1*) as described in both T-ALL patients and cell lines [47,48]. However, the leukemic role of *SIX6* in T-ALL remains unclear. 

### 2.4. Deregulated NKL Homeobox Genes in T-Cell Lymphoma

The oncogenic role of NKL homeobox genes was first described in T-ALL. Today, these genes are reportedly deregulated in all analyzed types of hematopoietic malignancies [24]. In the following, I summarize studies describing normal and aberrant activities of NKL homeobox genes which also play a role in T-cell lymphomas. 

#### 2.4.1. DLX

The human genome contains six Distal-less homeobox genes (DLX) which are arranged as neighboring pairs, sharing intergenic regulatory sites: *DLX1* and *DLX2*, *DLX3* and *DLX4*, *DLX5* and *DLX6* [69]. According to our NKL-code data, *DLX2* represents the only hematopoietically expressed DLX gene in humans [24]. It is physiologically expressed just in mature myeloid cells, including mast cells, monocytes and monocyte-derived DCs [22]. *DLX2* is located at chromosomal position 2q31 next to its paralogue gene *DLX1* which is normally silent in hematopoietic cells. However, expression of *DLX1*, *DLX2* and *DLX3* was reported in developing NK-cells of mice [70]. This particular stage was not included in the established NKL-code, requiring additional examinations in humans. Aberrant DLX gene expression has been shown for both *DLX1* and *DLX2* in T-ALL, AML and myelodysplastic syndrome (MDS) patients, showing divergent regulation in the malignant context [18,22]. In AML, oncogenic ERK-signalling drives the expression of both *DLX1* and *DLX2* [71]. In murine T-cell lymphoma, aberrant expression of neighboring *DLX5* and *DLX6* was correlated with inversion of chromosome 6, juxtaposing the loci of these NKL homeobox genes with T-cell receptor gene *Tcrb* [72]. *DLX5* expression was also detected in primary human T-cell lymphoma samples while *DLX6* tested negative [72]. Functionally, *DLX5* promotes NOTCH-signalling and the AKT-pathway in a mouse model which may represent its main oncogenic function [73,74]. 

#### 2.4.2. HHEX

Hematopoietically expressed homeobox gene (*HHEX*) is overexpressed in subsets of all analyzed T-cell malignancies [18,44]. However, our recent study demonstrates that *HHEX* acts like a tumor suppressor gene in ALCL cell lines. *HHEX* inhibits apoptosis and supports ILC3 differentiation. Potential target genes in these contexts are *CASP8*, *FOXO3* and *BHLHE40*, respectively [21]. Of note, ILC3 physiologically expresses *HHEX* and may represent a cell of origin for ALCL [21,75]. Thus, *HHEX* may act as an oncogene or tumor suppressor gene in T-cell lymphomas. 

#### 2.4.3. HLX

H2.0 like homeobox (*HLX*) is overexpressed in several hematopoietic malignancies including ALCL, DLBCL and HL [19,21]. Aberrant activation by STAT3 plays a dominant role in *HLX* expression in these tumor types and represents a hallmark factor in ALCL [76]. Analyses of several ALCL cell lines revealed genomic gains of the *STAT3* and *HLX* loci and demonstrated a direct activating impact of STAT3 in HLX expression. These data highlight *HLX* as an important oncogene and STAT3-target in this malignancy [21]. The requirement of the nuclear localization of STAT3 for HLX activation has been shown in HL cell line L-540 [77]. The role of Epstein–Barr virus mediated activation of STAT3 in *HLX* expression was demonstrated in DLBCL cell line DOHH-2 [78]. Thus, several oncogenic mechanisms activating *STAT3* and *HLX* have been described in hematopoietic malignancies. 

#### 2.4.4. MSX1

Muscle segment homeobox gene 1 (*MSX1*) is aberrantly expressed in several types of leukemia and lymphoma, including ALCL, HL, HSTL, mantle cell lymphoma (MCL) and T-ALL [18,19,79]. In the hematopoietic system, *MSX1* is normally expressed in the CLP and mature NK-cells [18,20]. These expression patterns reflect its contrasting roles as oncogene in T-ALL and tumor suppressor in NK-cell leukemia [20,45]. In T-ALL, downregulated BMP-signalling pathway and upregulated chromatin-mediator AUTS2 have been reported as activating mechanisms for *MSX1* expression [44,45,80]. In HSTL, *MSX1* is activated by AUTS2 and PDGF-signalling, and repressed by BMP4-signalling. The latter pathway is inhibited via PDGF-signalling, thereby contributing to *MSX1* activation [44]. Finally, *MSX1* plays a major role in generation and development of neural crest cells [81], supporting its role as lineage regulator in stem/progenitor cells.

#### 2.4.5. NANOG

NANOG is a stem cell factor in hematopoiesis as well as in the development of other tissue types [18,22,82]. In a transgenic mouse model, Das and colleagues showed that NANOG supported the oncogenic potential of MYC in the generation of T-cell lymphoma [83]. MYC in turn promotes the expression of *NANOG*, and both factors coregulate together with SOX2 particular target genes to drive self-renewal [83]. 

#### 2.4.6. NKX2-2

*NKX2-2* is ectopically expressed in several types of T-cell lymphoma including PTCL, AITL, ALCL and ATLL [X]. In HSTL cell lines DERL-2 and DERL-7 *NKX2-2* is mutually activated by *MSX1* [44]. *NKX2-2* and the closely related *NKX2-1* are aberrantly activated by chromosomal translocations in T-ALL while in DLBCL *NKX2-1* expression is driven by an altered chromatin configuration [61,63]. Downstream activities have been analyzed in T-ALL, showing that NKX2-2, like NKX2-1 and NKX2-5, activate expression of the hematopoietic TF *MEF2C* [60,61]. Normally, *NKX2-2* plays a role in the development of the brain and pancreas but is absent from hematopoietic cells and tissues [84,85]. 

Additional NKL homeobox genes deregulated in T-cell lymphoma patients are *NKX2-3*, *NKX3-1*, *EN1*, *EN2*, *HMX2*, *NKX1-1*, *NKX2-1*, *NKX2-5*, *NKX6-1*, *TLX1*, *TLX3* and *VAX2* [44]. To date, these genes have not been analyzed in more detail in this group of hematopoietic malignancies. Nevertheless, analyzed and described examples show that deregulated NKL homeobox genes play basic pathogenic roles in T-cell lymphomas, performing various oncogenic functions. Deregulating mechanisms reported in leukemia/lymphoma include chromosomal translocation, chromatin modifications, mutations in TF binding sites, altered DNA-methylation, and aberrant activities of particular TFs and signalling pathways like BMP, ERK, or NOTCH. Downstream effects of these genes are even more divers, including activation of specific TFs and micro RNAs, inhibitory interaction with cell type specific TFs, deregulation of differentiation markers and signalling pathways, in addition to disturbed proliferation, apoptosis and differentiation [24]. Notably, ectopically expressed NKL homeobox genes highlight the role of activated alternative developmental processes for this group of oncogenes. The NKL-code and its oncogenic violation discloses an underlying pathological principle and the list of oncogenic activities performed by deregulated NKL homeobox genes comprises all characteristics described for tumorigenesis [24].

### 2.5. Tumor Cell Lines as Models for Deregulated NKL Homeobox Genes

Tumor cell lines are clonal cells that grow unlimited in culture. They are genomically stable, survive freezing and thawing, and are available for all investigators from cell line banks. Their genomic, epigenomic and transcriptional characteristics correlate to their cell of origin. Therefore, tumor cell lines represent suitable experimental models for that tumor type from which they were derived. Therefore, it is of fundamental importance to use authenticated and well-characterized cell lines to be able to transfer cell line data to particular cancers. In the literature evaluated and systematically listed hematopoietic cell lines have been described which meet these criteria and include the T-cell lymphoma type ALCL in addition to other tumor entities like AML, chronic myeloid leukemia, B-cell precursor ALL, double-hit B-cell lymphomas, HL, MDS, multiple myeloma, NK-cell leukemia, primary effusion lymphoma and primary mediastinal B-cell lymphoma [86,87,88,89,90,91,92,93,94,95,96,97]. Recently, we have generated exomes and transcriptomes for 100 leukemia/lymphoma cell lines, providing highly comparative sequence data for a large panel of hematopoietic tumor entities including T-cell malignancies [43]. These data may help to identify novel genes and their regulatory connections involved in the pathogenesis of particular tumor types.

Deregulated NKL homeobox genes were usually first ascertained in patients and subsequently investigated experimentally in cell lines to reveal mechanisms of deregulation and downstream activities. Table 1 shows T-cell lines which aberrantly express particular NKL homeobox genes. They may serve as models to study upstream and downstream factors of those deregulated NKL homeo-oncogenes. To find novel models comprehensive expression data are required generated by gene expression profiling or RNA-sequencing of cell lines. Comparison with the NKL-code reveals ectopic activities of NKL homeobox genes. Aberrant expression of NKL-code members depends on the origin (lineage, stage) of the particular cell line. Overexpressed NKL-code members in accordance with their stage-specificity deserve corresponding controls for identification. Generally, there is a need for the establishment of additional hematopoietic cell lines including T-cell lines to cover described subtypes and variants of the tumors.

## 3. Conclusions

In summary, NKL homeobox genes are physiologically expressed in hematopoiesis in a unique and specific pattern which we have termed NKL-code. This code serves to identify and evaluate deregulated NKL homeobox genes in leukemia/lymphoma. Aberrant activities of these basic developmental regulators contribute to the generation of T-cell malignancies. The knowledge of their pathophysiological activity may support the design of improved diagnostics and novel therapies in the future. However, direct pharmacological targeting of TFs is generally difficult. Moreover, NKL homeobox genes impact fundamental processes in various tissues, anticipating strong side effects after treatment. Validated tumor cell lines represent suitable models to study the regulation and function of NKL homeobox genes in normal and malignant T-cells and may help to uncover suitable therapeutic targets.
